# Does Altered Membrane Glycosylation Contribute to Neurodevelopmental Dysfunction in Autism Spectrum Disorder?

**DOI:** 10.3390/membranes16010018

**Published:** 2026-01-01

**Authors:** Vinicius J. S. Osterne, Messias V. Oliveira, Vanir R. Pinto-Junior, Francisco S. B. Mota, Benildo S. Cavada, Kyria S. Nascimento

**Affiliations:** 1 BioMol-Lab, Department of Biochemistry and Molecular Biology, Federal University of Ceará, Fortaleza 60440-970, Ceará, Brazil; mvoliveira@alu.ufc.br (M.V.O.); juniorreis@alu.ufc.br (V.R.P.-J.); bscavada@ufc.br (B.S.C.); 2 IPREDE—Instituto da Primeira Infância, Fortaleza 60821-728, Ceará, Brazil; sulivan.mota@iprede.org.br

**Keywords:** glycoconjugates, sialic acid, high-mannose, lipid rafts, synaptic plasticity

## Abstract

Neuronal development relies on cell-surface glycoconjugates that function as complex bioinformational codes. Recently, altered glycosylation has emerged as a central mechanistic theme in the pathophysiology of autism spectrum disorder (ASD). Critically, the brain maintains a distinctively restricted glycan profile through strict biosynthetic regulation, creating a specialized landscape highly susceptible to homeostatic perturbation. This “membrane-centric vulnerability” spans both glycoproteins and glycolipids; however, evidence remains fragmented, obscuring their pathogenic interplay. To bridge this gap, this review synthesizes evidence for these two primary classes of membrane glycoconjugates into a unified framework. We examine how defects in key glycoproteins (such as NCAM1 and neuroligins) directly impair synaptic signaling, trafficking, and plasticity. We then demonstrate how these defects are functionally coupled to the glycolipid (ganglioside) environment, which organizes the lipid raft platforms essential for glycoprotein function. We propose that these two systems are not independent but represent a final common pathway for diverse etiological drivers. Genetic variants (e.g., MAN2A2), environmental factors (e.g., valproic acid), and epigenetic dysregulation (e.g., miRNAs) all converge on this mechanism of impaired glycan maturation. This model elucidates how distinct upstream causes can produce a common downstream synaptic pathology by compromising the integrity of the membrane signaling platform.

## 1. Introduction

Neuronal development depends on the precise orchestration of molecular interactions at the cell surface. Here, glycoconjugates (glycoproteins and glycolipids) govern adhesion, migration, differentiation, and the establishment of synaptic networks [1]. Glycans serve as complex bioinformational codes that mediate recognition and signaling, ultimately defining neuronal identity and connectivity [2,3,4]. In recent years, altered glycosylation has emerged as a relatively new mechanistic axis in neurodevelopmental disorders, with particularly strong evidence in autism spectrum disorder (ASD) [5]. Clinical and multi-omic analyses have revealed recurrent remodeling of the neuronal glycome and implicated several glycosylation-related genes in disease risk and phenotypic variability [6]. Mammalian brain glycoproteins display a simplified glycan profile compared to other tissues, a feature hypothesized to arise from tight transcriptional regulation rather than deficient synthetic capacity [7]. Consequently, disruptions to this strictly regulated machinery can have profound effects on neural function.

Although glycosylation is highly prevalent among secreted and membrane proteins, its biological implications are especially pronounced in the neuronal membrane, where topology and microenvironment define specialized cellular functions. As noted by Michalak et al. (2021), “membrane proteins are frequently highly glycosylated, which is both linked to physiological processes and of high relevance in various disease mechanisms” [8]. Membrane glycosylation regulates solubility, charge, stability, and resistance to proteolysis while organizing lipid rafts that coordinate adhesion, signaling, and endocytosis [9]. Recent proteomic mapping has shown that the vast majority of glycosylated proteins in the brain localize to the plasma membrane or extracellular compartments, highlighting the topographic specificity of this process [10]. This localized view is particularly relevant to ASD, where disruptions in glycosylation within discrete membrane domains may drive pathological changes in connectivity.

This membrane-centric vulnerability in ASD involves two primary classes of molecules. First, glycolipids, such as the sialic acid-containing gangliosides, are essential for organizing cholesterol-rich microdomains. These domains regulate receptor signaling and synapse formation [11,12,13,14]. Second, glycoproteins, which are critical for fine-tuning neuronal polarity, adhesion, and signal integration. These include synaptic adhesion proteins such as neurexins and neuroligins, whose trafficking and function depend on sequential N- and O-glycosylation [9,15]. However, evidence for these distinct molecular systems often remains fragmented, obscuring the connections between them.

Thus, the primary goal of this review is to synthesize these disparate findings into a unified document. We will first examine the distinct roles of membrane-bound glycoconjugates, focusing on key synaptic glycoproteins (NCAM, neuroligins, glypicans) in neural circuit formation followed by glycolipids (gangliosides). We then connect these protein-level disruptions to the underlying genetic and epigenetic vulnerabilities that compromise the glycosylation machinery, including evidence from Congenital Disorders of Glycosylation (CDGs). Finally, we propose a convergent model where these diverse factors lead to a final common pathway of glycan-mediated membrane dysfunction, driving the synaptic and connectivity deficits characteristic of ASD.

## 2. Membrane Glycoproteins and Neurodevelopment

**Figure 1:**
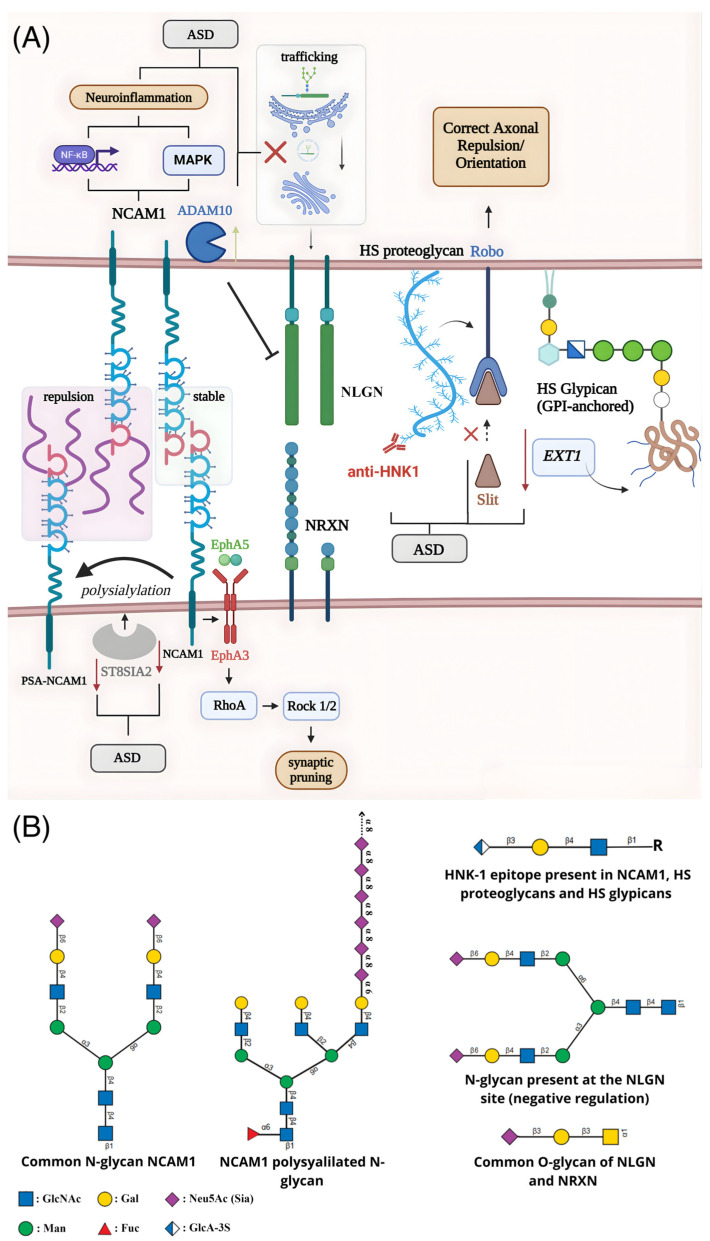
Schematic representation of alterations in glycosylation and their impact on synaptic dysfunction associated with ASD. (**A**) The enzyme ST8SIA2 catalyzes the polysialylation of NCAM1, modulating its adhesive function, axonal dynamics, and synaptic remodeling. Beyond cell adhesion, NCAM1 forms a complex with EphA3, whose activation by EphrinA5 triggers the RhoA–Rock1/2 pathway and promotes axonal retraction and synaptic pruning. NCAM1 also influences neuroinflammatory responses by modulating NF-κB and MAPK signaling in astrocytes. Reduced expression of NCAM1 and ST8SIA2 in ASD diminishes polysialylation and disrupts synaptic plasticity and stability. Glycosylation defects also impair neuroligin (NLGN), where mutations hinder ER-to-Golgi trafficking or enhance ADAM10-mediated cleavage, reducing functional NLGN at the synaptic membrane. Loss of the HNK-1 epitope on specific proteoglycans interferes with Slit/Robo signaling, compromising axonal guidance, while anti-HNK-1 antibodies may further disrupt the blood–brain barrier and extracellular matrix interactions. Decreased EXT1 expression impairs heparan sulfate (HS) chain elongation in glypicans, adding further deficits to axonal navigation and circuit formation during neurodevelopment. (**B**) Representative glycan structures associated with these mechanisms, including N-glycans and polysialylated chains present in NCAM1, O-glycans characteristic of neuroligin and neurexin, and HS/heparan sulfate motifs found in proteoglycans and glypicans.

The model centered on membrane-specific dysfunction is perhaps most clearly illustrated by the synaptic glycoproteins. These molecules, including critical cell adhesion molecules (CAMs) and receptors, rely on their glycan moieties for proper folding and function modulation, which is important for trafficking, binding affinity, and signaling properties (Figure 1). A failure in this system, therefore, provides a direct, mechanistic link from a specific glycan defect to a functional synaptic impairment. This section will review the evidence for several key glycoprotein families and glycan epitopes implicated in ASD.

### 2.1. NCAM1 and Polysialylation (PSA) as Regulators of Adhesion and Plasticity

NCAM1 (Neural Cell Adhesion Molecule) is a glycoprotein predominantly expressed on the surface of nerve cells, acting in cell adhesion within the developmental and plasticity pathways of the nervous system, and regulating the formation, maturation, and function of synapses [16,17,18]. Its adhesive function, including homophilic adhesion between NCAM1 molecules in adjacent cells, is regulated by the post-translational modification that is the addition of polysialic acid (PSA). PSA is an extensive, negatively charged glycan that acts as a steric modulator of NCAM1, functioning as an “anti-adhesive” molecule that promotes steric and electrostatic repulsion [19,20]. The presence of PSA (forming PSA-NCAM) decreases intermembrane adhesion, facilitating the structural plasticity necessary for neuronal migration, axonal growth, and synaptic remodeling during neurodevelopment. When PSA is removed, NCAM1 promotes strong adhesion, stabilizing synapses [21,22]. As the brain matures, PSA levels decrease, NCAM1 becomes more adhesive, and neural circuits stabilize. The enzymes responsible for adding PSA to NCAM1 are two polysialyltransferases (polySTs): ST8SIA2 (ST8SiaII or STX) and ST8SIA4 (ST8SiaIV or PST). Both are capable of adding PSA to NCAM1, but ST8SIA2 is expressed at high levels during embryonic and postnatal development, while ST8SIA4 is predominantly expressed in adulthood [20,23,24,25]. In addition to its role in cell adhesion, NCAM1 actively participates in synaptic pruning, forming a complex with the EphA3 receptor which, upon binding to the EphrinA5 ligand, activates the RhoA-Rock1/2 intracellular signaling pathway to induce axonal retraction [26,27]. NCAM1 also contributes to synaptic stabilization by anchoring to the cytoskeleton via Ankyrin, and modulates neuroinflammatory processes by regulating NF-κB and MAPK pathways in astrocytes [28,29].

Alterations in these functions are directly associated with ASD. In children with ASD, the regulation of neuroplasticity is compromised, with significantly lower plasma levels of the NCAM1 protein. These reduced levels correlate negatively with social communication skills and social motivation, but positively with gross motor skills and the developmental quotient [30]. At the genetic level, the ST8SIA2 gene is identified as a susceptibility factor for ASD. Children with ASD exhibit lower levels of ST8SIA2 expression, which correlates with more severe stereotyped behaviors and poorer daily living skills [31,32]. This combined deficiency of NCAM1 and PSA-NCAM suggests a failure in the mechanisms of synaptic plasticity and pruning. Animal models deficient in ST8SIA2 exhibit core ASD behaviors [33]. NCAM1 dysfunction is also linked to the neuroinflammation observed in ASD, through its failure to regulate astrocyte proliferation and inflammatory signaling [29,34].

PSA-NCAM also plays a role in the balance between excitatory (glutamate) and inhibitory (γ-aminobutyric acid, GABA) neurotransmission, a mechanism associated with the ASD pathogenesis. PSA-NCAM molecularly regulates both sides of this balance. On the excitatory side, glycosylation is essential for the trafficking and sensitivity of glutamate receptors (NMDA and AMPA). It regulates synaptic plasticity largely by restricting signaling via NMDA receptors [35]. On the inhibitory side, PSA-NCAM regulates GABAergic synapses, helping to mediate plasticity, restricting excessive innervation of interneurons, and actively participating in the pruning of inhibitory terminals [27,36,37].

In summary, in children with ASD, neuroplasticity regulation is compromised due to significantly lower levels of the NCAM1 protein and reduced expression of the ST8SIA2 enzyme, which produces PSA-NCAM. This combined deficiency suggests a failure in PSA-mediated neuroplasticity during neurodevelopment, potentially resulting in the core behaviors of ASD [30,33,34]. The functional consequence of low PSA-NCAM levels is that brain connections can become prematurely stable [20,37]. This directly leads to a failure in synaptic pruning, where the brain loses the ability to eliminate inefficient or incorrect connections. This failure in circuit remodeling is consistent with the pattern of local hyperconnectivity and long-distance hypoconnectivity observed in ASD. At the behavioral level, this molecular profile provides a biological basis for explaining cognitive rigidity and repetitive behaviors, suggesting that the brain is unable to adapt or reconfigure its circuits flexibly [20,22,38,39].

### 2.2. The Neuroligin–Neurexin System and Its Dependence on N- and O-Glycosylation for Trafficking and Binding

Neuroligins (NLGNs) and neurexins (NRXNs) are families of cell adhesion molecules (CAMs) critical to synaptic architecture. NRXNs are located on the presynaptic membrane, while NLGNs are located on the postsynaptic membrane. Their mechanism involves the formation of a trans-synaptic bridge. The extracellular domain of NLGN (similar to acetylcholinesterase) binds to the extracellular LNS domains of NRXN in a calcium-dependent interaction [40]. This bidirectional binding acts as a fundamental organizing mechanism for the formation, maturation, stability, and function of synapses. The NRXN-NLGN system directly regulates synaptic transmission and plasticity; its immense diversity of isoforms, generated by alternative splicing, creates a molecular code that specifies synapse type (excitatory or inhibitory) and neuronal circuit connectivity [40,41].

Glycosylation is an extensive and fundamental post-translational modification that regulates the NLGN-NRXN system at multiple levels, dictating both its transport to the synapse and its specific binding capacity [42,43]. For NLGNs, N-glycosylation is the dominant event and an absolute requirement for transport. The glycosylation profile of NLGNs begins in the Endoplasmic Reticulum (ER), where they receive immature high-mannose N-glycans. This step is essential for correct protein folding and serves as a quality control point [44,45]. If N-glycosylation fails, or if ASD-associated mutations (such as R451C (NLGN3) or R87W (NLGN4X)) cause incorrect folding, the protein is recognized as defective and is retained in the ER and Golgi [46]. This prevents NLGN from being transported to the cell surface and accumulating in the synapse, resulting in a loss of function. A notable example is the R101Q mutation (NLGN4X), associated with autism, which specifically prevents N-glycosylation at the adjacent, conserved N102 site, directly leading to protein retention in the ER and Golgi apparatus. Equivalent mutations to R101 in NLGN1, NLGN2, and NLGN3 also impair maturation and transport [45,47].

As mature NLGNs move through the Golgi apparatus, their N-glycans are processed into “hybrid” and “complex” forms, which may include sialic acid [6,43,45]. N-glycosylation also modulates binding. Alternative splicing at the B Site (SSB) of NLGN1 introduces an N-glycosylation site (N303). The presence of this specific glycan decreases binding affinity to β-neurexins and, notably, prevents NLGN1 from inducing inhibitory synapses [40,43,48,49,50]. Conversely, splicing at the A Site (SSA) of NLGN1 increases its binding affinity to Heparan Sulfate (HS) present in neurexins [48,51,52]. In addition to N-glycosylation, NLGNs also possess O-glycosylation in their stem regions, but its impact on transport and function is unclear [43,45,53].

For NRXNs, glycosylation is central to binding and specificity [51]. Their profile is characterized by extensive O-glycosylation modifications in their stems and, most notably, by being Heparan Sulfate proteoglycans (HSPGs) [48,54,55]. The HS chains act as co-receptors that stabilize high-affinity interaction with canonical partners, such as NLGN1, and are absolutely essential for binding to non-canonical partners [56]. Alternative splicing of NRXN1 at Site 5 (S5) directly regulates this profile, as the inclusion of the S5 insert confers an additional site for HS binding, increasing the HS valence of the protein. Functionally, this higher HS valence is linked to reduced NRXN1 protein levels and lower neurotransmitter release [57].

Mutations in NLGN4X (such as G84R, R87W, G99S) cause loss of function by ER retention due to misfolding and defective glycosylation. The L593F variant causes loss of function by an opposite mechanism: it accelerates the proteolytic cleavage of mature NLGN4X on the cell surface by the protease ADAM10, reducing the amount of functional NLGN4X in the synapse. Inhibition of transport by misfolding variants (except for R87W) could be pharmacologically rescued in cell culture using chemical chaperones (such as 4PBA), which restores protein maturation and its synaptogenic function [44].

Failures in synaptic protein transport and the resulting dysregulation of synaptic strength due to aberrant glycosylations have been associated with ASD-related behaviors. This transport failure results in a loss of function, with drastically reduced levels of functional NLGN on the cell surface, impairing synaptogenesis and leading to synaptic dysfunction, such as increased excitatory transmission. HS glycosylation of NRXNs is also implicated, as regulation of HS levels has been shown to directly affect ASD-associated behaviors in mice, such as ultrasonic vocalizations (communication) and grooming (repetitive behaviors) [54,58].

### 2.3. Human Natural Killer-1 (HNK-1)

The HNK-1 (human natural killer-1) carbohydrate epitope is a unique sulfated trisaccharide structure characterized by a 3-sulfoglucuronyl residue linked to lactosamine structures (Galβ1-4GlcNAc) [59]. This sequence is present in several classes of glycoconjugates found in nervous system cells [59]. In the brain, HNK-1 expression is finely regulated and plays essential roles in neurodevelopment, mediating cell–matrix interactions, cell–cell adhesion, neural-glial adhesion, synaptic plasticity, spatial learning, and memory [60]. It is directly involved in the determination, growth, and regeneration of axons, and its presence modifies N-glycans of various neural cell adhesion molecules, such as N-CAM, L1, myelin-associated glycoproteins, and the glutamate receptor GluA2 [59,61].

The relevance of HNK-1 to ASD is evidenced by brain abnormalities that significantly overlap with those found in the disorder [59]. ASD is frequently characterized by structural abnormalities, such as an increased number of dendritic spines and decreased long-distance connections [62,63]. Animal models lacking the key enzymes for HNK-1 biosynthesis (such as GlcAT-P or HNK1-ST sulfotransferase) demonstrate significant deficits in synaptic plasticity and spatial learning, mimicking functional aspects of ASD [64,65]. One of the mechanisms linking HNK-1 to these structural defects is the Slit/Robo signaling pathway. This pathway acts on the correct orientation of axons, and its dysregulation is associated with ASD. HNK-1 is expressed in HS proteoglycans, and HS is a direct modulator of the Slit/Robo interaction. In the absence of HS, the repulsive activity of the Slit protein is lost, resulting in excessive dendritic spine formation, which aligns with the structural phenotype observed in ASD [63,66].

In addition to its direct brain function, HNK-1 is expressed in the extravillous trophoblast of the placenta, the tissue that interacts directly with the maternal immune system [59]. HNK-1 is known to be highly immunogenic, and one hypothesis in ASD pathogenesis involves maternal immune activation leading to defects in these maternal-fetal interactions. It is proposed that immunogenic reactions may target the regulation of HNK-1 expression in the trophoblast [67,68]. The generation of anti-HNK-1 antibodies can lead to various neurological damage, disrupt the blood–brain barrier, and inhibit neural crest migration by disrupting the binding of cells to laminin in the extracellular matrix [69,70].

HNK-1 also acts on glutamatergic and GABAergic circuits that are out of balance in ASD [59]. The epitope is specifically present in inhibitory interneurons that express parvalbumin and is found in GABAergic and glutamatergic axonal terminals in these cells. Its expression is even regulated by NMDA receptor activity [71,72]. Therefore, the dysregulation of HNK-1 expression, whether through axonal developmental pathways such as Slit/Robo or through immune processes at the maternal-fetal interface, is implicated in multiple central pathophysiological mechanisms of atypical neurodevelopment in ASD.

### 2.4. Glypicans Coordinating Synaptogenesis

Glypicans (GPCs) are a family of HS proteoglycans, comprising six members (GPC1 to GPC6) in mammals [73]. Structurally, they are defined by a core protein that anchors to the outer face of the cell membrane via a glycosylphosphatidylinositol (GPI) anchor [74]. However, their structure allows glypicans to exist both membrane-bound and in soluble form. The general role of glypicans in the nervous system is to act as central regulators and co-receptors in the organization of synapses. They function as essential molecular bridges that help establish, mature, and stabilize connections between presynaptic and postsynaptic neurons [51,75].

Different glypicans act at different stages of synaptic development. GPC4 and GPC5, frequently secreted by astrocytes, act in the initial formation of the synapse. They initiate synaptogenesis by promoting the recruitment of AMPA receptors containing the GluA1 subunit to the postsynaptic side [75,76]. GPC4 binds to presynaptic receptors and triggers the release of Neuronal Pentraxin 1 (NP1), which in turn clusters AMPA receptors [77]. In contrast, GPC5 (also derived from astrocytes) is more associated with the maturation and stabilization of formed synapses. GPC5 contributes to maturation by recruiting AMPA receptors containing the GLUA2 subunit in intracortical synapses [75]. In all these roles, the HS chains of glypicans are essential, as they mediate most of these interactions with other synapse-organizing proteins. This includes interactions with LRRTMs, leucine repeat-rich post-synaptic transmembrane proteins, and RPTPs, tyrosine protein receptor phosphatases, such as PTPδ and PTPσ, which are pre-synaptic proteins [51,74,75,78,79,80].

Given their role in organizing neural connections, glypican dysfunction is strongly implicated in neurodevelopmental disorders, including ASD [74]. Mutations in glypicans are associated with neurological disorders such as autism. Mutations in GPC4 regulatory regions in individuals with ASD decreased GPC4 expression [81]. Animal models reinforce this connection; for example, mice with GPC4 knockout exhibit ASD-like behaviors, such as deficits in social interaction [82].

The link to ASD is also associated with HS chains, the glycidic component of glypicans. Mutations in genes associated with HS chain anchoring (such as *EXT1*), responsible for HS deficiency in glypicans, have been found in patients with autism and mild intellectual disability [83]. This finding was reinforced in animal models, where mice exhibited ASD-like behaviors, such as sociocommunicative deficits and stereotyped behaviors [84]. Beyond synthesis, specific modification of these chains is also critical. Genetic variants (SNPs) associated with ASD have been identified near the *Hs3st-5* gene, an enzyme responsible for a specific sulfation pattern of HS chains [85]. This indicates that both the presence and precise chemical structure of HS chains in glypicans are necessary for their correct function. When this glycosylation is deficient or structurally aberrant, glypicans fail to organize synapses properly, contributing to ASD pathophysiology.

## 3. Membrane Glycolipids and Neural Circuit Formation

The preceding sections demonstrate how aberrant glycosylation of specific proteins can directly impair synaptic adhesion and signaling. However, these glycoproteins do not operate in isolation. Their function is critically dependent on their spatial organization and lateral mobility within the plasma membrane, a property dictated by the other major class of glycoconjugates: the glycolipids. As structural organizers of lipid rafts, the complex gangliosides GM1, GD1a, GD1b, and GT1b dominate the nervous system and are essential for the stability and function of these microdomains. Their amphiphilic nature enables interactions with both membrane lipids and extracellular proteins, defining the physicochemical identity of the neuronal glycocalyx [86]. Despite their negative charge, these gangliosides self-aggregate through the “NH trick,” a hydrogen-bonding mechanism between the carboxyl group of sialic acid and the amide of N-acetyl groups in neighboring sugars [87,88]. This induces cholesterol-stabilized nanocluster formation that nucleates membrane rafts, a property that simpler gangliosides such as GM3 and GM4 do not possess [89]. Molecular and biophysical analyses reveal that GM1 and related species generate heterogeneous raft zones with dense, ordered cores and more flexible peripheries [90,91]. Within neuronal membranes, these dynamic ganglioside–protein assemblies modulate receptor trafficking and synaptic vesicle fusion. They do this by stabilizing complexes such as synaptotagmin and SNARE proteins, which fine-tunes neurotransmitter release [92]. GM1-enriched rafts also interact with growth factor receptors, including EGF and PDGF, coordinating local signaling pathways that promote neuritogenesis and axonal guidance [93]. In parallel, GD1a and GT1b mediate axon–myelin stability through raft-associated recognition of myelin-associated glycoprotein (MAG) [86].

Genetic evidence from human studies confirms this critical dependence. Loss-of-function mutations in genes encoding key ganglioside-synthesizing enzymes, particularly ST3GAL5 (GM3 synthase) and B4GALNT1 (GM2/GD2 synthase), lead to severe neurodevelopmental disorders. ST3GAL5 mutations abolish the synthesis of GM3, the precursor of all complex brain gangliosides, causing profound intellectual disability, choreoathetosis, and neurocutaneous defects [94]. Biochemical and transcriptomic analyses of patient-derived fibroblasts confirmed the complete absence of GM3 and collateral changes in glycolipid and glycoprotein glycosylation, while morpholino knockdown of ST3GAL5 in zebrafish resulted in extensive apoptotic cell death in developing brain regions, reinforcing its essential role in neural development [95,96]. Similarly, B4GALNT1 mutations disrupt complex a- and b-series ganglioside biosynthesis, leading to hereditary spastic paraplegia with progressive central demyelination, axonal loss, and cognitive deficits [97,98].

In ASD, accumulating evidence points to altered sialylation and immune dysregulation involving these specific glycolipids. A case–control study involving 82 ASD children demonstrated significantly lower plasma sialic acid (SA) concentrations and a higher positivity rate for anti-GM1 ganglioside antibodies compared to controls [99]. The presence of anti-GM1 antibodies was associated with disease severity, suggesting a link between impaired sialylation, autoimmunity, and neurobehavioral phenotypes. This is significant, as gangliosides account for ~75% of the brain’s total SA. Supporting this, altered GM1 levels in cerebrospinal fluid and erythrocyte membranes of ASD patients have been reported [100,101,102]. Complementary evidence from a recent experimental study demonstrated that early-life SA supplementation in valproate-induced autism model rats significantly alleviated stereotyped behaviors and social deficits, improved hippocampal neuron morphology, and enhanced learning and memory [103]. These effects correlated with upregulation of GNE and ST8SIA2 (key SA synthesis genes) as well as increased serum BDNF levels, reinforcing the functional coupling between SA metabolism and neuroplasticity. Untargeted metabolomics further revealed SA-dependent modulation of amino acid, pyrimidine, and galactose pathways implicated in ASD, highlighting the systemic metabolic footprint of altered glycosylation. At the molecular level, imbalances in major gangliosides (GM1, GM3, GD1a, GT1b) and cholesterol content are central to altered lipid raft organization, dopaminergic signaling, and synaptic connectivity in ASD [100]. Altogether, this data collection indicates that altered sialic acid metabolism, aberrant ganglioside–cholesterol interactions, and anti-ganglioside autoimmunity represent interconnected pathways driving synaptic and neurodevelopmental abnormalities in autism.

## 4. Genetic and Epigenetic Disruption of Glycosylation Pathways in ASD

The hypothesis that aberrant glycosylation contributes to ASD is substantiated by evidence identifying the glycosylation machinery as a significant point of vulnerability. Table 1 contains a list of genes that encode glycoproteins or proteins related to glycosylation processes that are associated with ASD. This disruption is not typically characterized by the severe, multi-systemic presentations of classical CDGs. Instead, the evidence points toward polygenic risk, subtle “glycan susceptibility factors,” and environmental factors that collectively compromise the fidelity of glycan structures, particularly at the neural membrane [39].

### 4.1. Key Glycogene Variants as “Glycan Susceptibility Factors” in ASD

Large-scale genomic studies, including Genome-Wide Association Studies (GWAS) and Copy Number Variation (CNV) analyses, have identified numerous genetic variants in glycogenes [39]. This supports a polygenic risk model, exemplified by the initiation of mucin-type O-glycosylation, catalyzed by the polypeptide N-acetylgalactosaminyltransferase (GalNAc-T) family. GWAS and sequencing studies have highlighted *GALNT9* as associated with ASD [6,106]. Critically, *GALNT9* is specifically expressed in the brain (cerebellum, frontal lobe, spinal cord) with little expression elsewhere [107,108]. This tissue-specific expression explains how such a defect can result in a brain-specific phenotype rather than a multi-systemic CDG.

Evidence from N-glycan maturation pathways provides a similar molecular link. A recent case report described a child with ASD and intellectual disability carrying biallelic, loss-of-function mutations in *MAN2A2* (p.Arg560Gln and p.Gln1098Ter) [5]. *MAN2A2* encodes Golgi alpha-mannosidase II, an essential enzyme that trims N-glycans, facilitating their maturation from oligomannose/hybrid into complex-type structures [109]. Glycomic analysis of the patient’s serum confirmed a clear N-glycosylation defect: an accumulation of immature N-glycans and a reduction in fully sialylated complex N-glycans. Although the defect was systemic (detected in serum), the clinical phenotype was dominated by ASD and cognitive delay, not a classic, severe CDG [5]. Such findings extend the concept of CDGs into the autism realm as even partial disruptions of glycosylation enzymes can contribute to ASD or related phenotypes. In general, CDGs are proof of how vital glycosylation is for neurodevelopment: the majority of CDG patients present with severe neurological symptoms (hypotonia, seizures, developmental delays), and some exhibit autistic features. Notably, milder hypomorphic mutations in certain glycosylation genes may produce isolated neurodevelopmental issues such as ASD without the full multisystem CDG presentation [110]. This suggests the developing brain, which relies on an extraordinary density of complex-type N-glycans for synaptic adhesion molecules, is a “bottleneck” of glycosylation-dependent processes. The brain appears uniquely vulnerable to partial systemic disruptions in N-glycan maturation tolerated by other organs [111].

Evidence also points to other key nodes. Variants in *B4GALT1*, encoding a β-1,4-galactosyltransferase, have been identified as an ASD susceptibility gene, positioned as a central regulatory hub in gene networks [104]. Furthermore, the HS pathway is implicated through variants in *EXT1* (HS biosynthesis) and *GPC6* (Glypican-6), implicating the neural extracellular matrix (ECM) in axon guidance [112,113,114]. Finally, variants in *ST8SIA2*, the polyST responsible for synthesizing PSA on NCAM1 (as discussed in Section 2.1), are associated with ASD, directly implicating glycan-mediated plasticity [30,33,34]. Convergence also occurs from the opposite direction: mutations in high-confidence ASD genes that are *targets* of glycosylation. In these cases, the glycosylation machinery is intact, but the protein substrate is altered, phenocopying a primary glycosylation disorder. The most prominent example is the R101Q missense mutation in NLGN4, a high-confidence ASD risk gene [47]. As noted earlier, this mutation functions mechanistically as a glycosylation defect by abolishing the critical N-glycosylation site at N102. This single-site glycan loss causes the unglycosylated protein to misfold, be retained in the ER, and fail to traffic to the synaptic membrane, resulting in a synaptic loss-of-function [47]. Similarly, *NRXN1*, perhaps the highest-confidence ASD risk gene, is subject to critical HS O-glycan modifications, which dynamically regulate its binding to synaptic organizers and may influence trans-synaptic signaling by competing with other HSPGs like glypicans [55,57].

### 4.2. The VPA Rat Model and Epigenetic Regulation as a Convergent Mechanism

Evidence from environmental paradigms, such as the valproic acid (VPA) rat model, provides a critical parallel to these genetic disruptions [115]. Prenatal VPA exposure, which induces ASD-like behavioral phenotypes, offers a model for how an environmental effect can trigger a systemic collapse of the glycosylation machinery. Transcriptomic analysis of the medial prefrontal cortex (mPFC) in VPA-exposed rats identified 107 differentially expressed glycosylation-related genes, clustered in pathways essential for N-glycan core trimming, elongation, and ECM regulation [105]. This massive transcriptomic shift had a clear glycomic effect: lectin microarray analysis of the mPFC revealed a significant increase in high-mannose N-glycan signals and a decrease in α2,3-sialylated motifs [105]. This finding is a cornerstone of the hypothesis, as the glycomic signature of the environmental VPA model perfectly mirrors the N-glycan phenotype produced by the genetic *MAN2A2* loss-of-function [5,105]. This convergence strongly suggests that “impaired N-glycan maturation” represents a final common pathogenic pathway in ASD.

The widespread transcriptomic collapse observed in the VPA model suggests an epigenetic mechanism, specifically via microRNAs (miRNAs) with a compelling body of evidence indicating that specific miRNAs are consistently deregulated in ASD [116]. Recent analyses, such as the 2025 study by Mirabella and colleagues, demonstrated that these ASD-associated miRNAs have a high propensity to target the mRNAs of key glycogenes, providing a computational framework for this “epigenetic glycosylation signature” [104].

In this regulatory network, multiple interactions have been experimentally validated. Deregulated miRNAs in ASD patients target *DOLK* (Dolichol Kinase), essential for N-glycan precursor synthesis [117]. Another validated target is *EXT1* (Exostosin Glycosyltransferase 1), essential for HS biosynthesis; notably, conditional knockout mice lacking neuronal *EXT1* recapitulate the full spectrum of autistic symptoms [118]. Further targets include *GALNT2* and *B3GALNT2*, key O-glycosylation enzymes [119]. Network analysis also identified *B4GALT1*, itself an ASD susceptibility gene, as a central regulatory “hub” where multiple ASD-associated miRNAs converge [120]. This miRNA-mediated mechanism provides the missing link. It explains how environmental factors (like VPA) can alter a few key miRNAs. These miRNAs then simultaneously repress dozens of glycogenes, leading to catastrophic glycomic defects [104]. This positions the glycosylation machinery as a central, epigenetically sensitive hub where “nature” (genetic risk factors) and “nurture” (environmental factors) converge.

### 4.3. Convergence on Membrane-Specific Phenotypes

The initial connection between glycosylation and ASD was not derived from studies of autism but from clinical observations within the CDG patient population, where a subset of individuals exhibited ASD-like behaviors [121]. ASD is now recognized as a potential hallmark feature of certain CDGs, and the study of these rare, monogenic disorders provides a “map” toward the most critical glycosylation-dependent processes likely disrupted, albeit more subtly, in idiopathic ASD [6,121].

This distinction is critical, as the collective evidence in ASD does not point toward a global, systemic failure of all glycosylation, but rather a more subtle, brain-specific “glycan susceptibility” [39]. This “membrane glycosylation phenotype” is localized by several key factors: the brain-specific expression of key enzymes (like *GALNT9*), the unique vulnerability of the CNS to partial systemic enzyme defects (the *MAN2A2* case), and the focus on high-confidence ASD risk genes that function as points of glycosylation failure (like *NLGN4*). These distinct etiological pathways, spanning *MAN2A2* mutations, *NLGN4* mutations, VPA teratogenesis, and epigenetic miRNA-mediated repression, represent a pathogenic nexus. They all converge on a final common molecular pathology: a reduced concentration of mature, complex N-glycans, altered O-glycan profiles, and aberrant sialylation and glycosaminoglycan structures.

## 5. Glycan-Mediated Membrane Dysfunction as a Pathogenic Mechanism

The genetic and epigenetic disruptions detailed in Section 4 establish that the “glycocode” is a point of failure in ASD. This section addresses the functional consequences: how these aberrant glycan structures on the neuronal membrane lead to the synaptic dysfunction, altered connectivity, and circuit malformation characteristic of ASD.

### 5.1. Aberrant Glycosylation and the Disruption of Synaptic Function

Perturbations in glycosylation disrupt both the trafficking of membrane proteins and the interactions they mediate. The first domain of dysfunction lies in protein trafficking. N-glycans serve as quality-control tags in the ER. As previously detailed, the ASD-associated *NLGN4* R101Q mutation eliminates the N102 glycosylation site, causing misfolding, ER retention, and total loss of synaptic localization [47].

The second domain involves cell adhesion and plasticity. Polysialic acid (PSA) chains on NCAM1 act as anti-adhesive modulators [30]. Defects in *ST8SIA2* [33] or the hypo-sialylation observed in the VPA model [105] reduce PSA levels and cause hyper-adhesion, restricting neuronal mobility and network plasticity. Similarly, loss of the HNK-1 carbohydrate epitope impairs learning and memory in mouse models [65].

The third domain encompasses signal transduction and circuit organization. Glycans such as heparan sulfate (HS) act as essential co-factors. The Slit–Robo pathway, crucial for axon guidance, absolutely depends on HSPGs like glypicans to present Slit to its receptor [55,122]. Loss of HS disrupts axon repulsion, leading to aberrant synaptic wiring [123]. Likewise, Notch receptor activation depends on O-fucosylation and O-glucosylation, and its dysregulation has been linked to abnormal neurogenesis in ASD models [124].

At the systems level, these molecular perturbations converge on E/I imbalance, a core feature of ASD. The *NLGN4* R101Q mutation, for example, enhances excitatory transmission in human iPSC-derived neurons. Moreover, the general hypo-sialylation observed in ASD models alters voltage-gated ion channel gating, directly influencing neuronal excitability. Reduced sialylation may also impair neuron–microglia signaling by weakening sialic acid–Siglec “self” recognition, leading to excessive synaptic pruning [125,126]. Thus, defective glycosylation provides a unified molecular basis for the structural and functional abnormalities underlying ASD.

### 5.2. Potential Cross-Talk Between Glycoprotein and Glycolipid Domains

Neuronal membrane function is governed by a dynamic interplay between glycoproteins and the glycolipid-enriched microdomains. Recent analysis proposes a “dual regulator” model, in which gangliosides (like GM1) and cholesterol form a geometric and molecular coupling. In this model, gangliosides influence cholesterol distribution and chaperone the conformation of transmembrane proteins [100]. This protein-lipid raft coupling creates a critical “two-hit” vulnerability: synaptic integrity can be disrupted by a defect in the glycoprotein itself (the *NLGN4* mutation) or by a defect in the glycolipid raft it depends on [127]. Evidence indicates ASD is linked to both. In addition to the glycoprotein defects, multiple studies report ganglioside metabolism defects in ASD, including reduced plasma sialic acid and anti-ganglioside antibodies [99]. Animal models lacking complex gangliosides (*St3gal5* knockouts) exhibit disrupted lipid rafts and ASD-like behaviors [128]. This reveals a devastating potential for convergence: a systemic hypo-sialylation defect, as seen in the VPA model, could launch a dual-front assault on the synapse, simultaneously impairing glycoproteins (like PSA-NCAM) and glycolipids (gangliosides), leading to a catastrophic failure of the entire membrane signaling platform.

## 6. Glycoconjugates in Diagnosis and Therapeutic Strategies for ASD

### 6.1. Glycomic Profiling as a Diagnostic Tool

The search for objective, biological markers for ASD is a major priority, and analyzing the “glycome” in accessible biofluids represents a promising strategy. Table 2 presents potential biomarkers related to glycosylations. Studies using lectin microarrays, which employ high-affinity glycan-binding proteins, have identified distinct alterations in the serum glycopatterns of individuals with ASD. One of the most consistent findings was a marked increase in structures containing Siaα2-3Gal/GalNAc, a clear indication of altered sialylation [105,129].

A distinct but related line of inquiry has focused on markers of non-enzymatic glycation and oxidative stress. A blood test has been developed based on a panel of advanced glycation end-products, including Nε-carboxymethyl-lysine, Nω-carboxymethyl-arginine, and 3-deoxyglucosone-derived hydroimidazolone, along with the oxidative damage marker o,o’-dityrosine (DT). This panel could distinguish ASD from neurotypical development with high accuracy. Critically, ASD severity correlated positively with glycation adducts derived from methylglyoxal (MG-H1 and CEL) [131,132]. The divergence between enzymatic glycosylation (an enzymatic “hardware” problem) and non-enzymatic glycation (a metabolic “software” problem) is mechanistically important. A comprehensive biomarker panel may need to measure both to stratify patients. While blood is accessible, cerebrospinal fluid (CSF) may also be adequate for reflecting CNS pathology, but its use is hampered by low protein concentrations. However, advances in highly sensitive mass spectrometry are beginning to unlock the potential of the CSF glycome [129].

### 6.2. Emerging Therapeutic Avenues

While ASD is increasingly understood as a form of neurodiversity, interventions for co-occurring challenges are sought. Currently, no pharmacological treatments address the core symptoms. The research into glycosylation illuminates novel conceptual avenues rather than an immediate “cure.” Directly “fixing” the glycosylation process is an immense challenge; therefore, the more probable path involves targeting specific downstream effects. Direct Sialic Acid (SA) supplementation has been tested preclinically in the VPA-induced rat model, where it successfully ameliorated ASD-like phenotypes and normalized hippocampal neuron morphology [103]. A more nuanced strategy involves targeting the Sialic Acid-SIGLEC axis. In the CNS, sialic acids on neuronal membranes act as “checkpoint inhibitors,” binding to SIGLEC receptors on microglia [133]. The hypo-sialylation state in ASD may remove this inhibitory “brake,” contributing to microglial over-activation. Developing SIGLEC-agonists represents a novel anti-neuroinflammatory strategy [133].

A second avenue, based on the “dual regulator” model (Section 5.2), is the stabilization of lipid rafts. Interventions that modulate the cholesterol pathway or supplement specific gangliosides could stabilize these critical signaling platforms [134]. Broader downstream strategies include modulating epigenetic regulators (e.g., anti-miRNA oligonucleotides) [135,136], targeting metabolic stress (e.g., anti-glycating agents like carnosine) [137], or targeting specific neuroinflammatory and signaling cascades (e.g., mTOR, PI3K-Akt) [138].

It must be emphasized, however, that these therapeutic avenues remain highly conceptual. The value of the data presented in this review lies primarily in its power to elucidate the fundamental biology of membrane dysfunction in ASD. This mechanistic understanding, while essential, does not yet translate into an immediate or clear-cut therapeutic pipeline. These approaches are foundational, highlighting the complexity of the challenge while simultaneously revealing novel, albeit distant, targets for future investigation.

## 7. Conclusions

Altered membrane glycosylation is emerging as a critical convergent point in ASD, where defects ranging from ganglioside imbalances to miRNA dysregulation disrupt the synaptic architecture necessary for circuit refinement. The precise interplay between glycoproteins and membrane dynamics is still unclear. However, advances in high-throughput glycoprofiling and engineered lectins now allow far more precise interrogation of these mechanisms. By applying these tools to bridge the gaps between structural glycomics and synaptic physiology, future research can validate these molecular features as reliable biomarkers and targets for therapeutic intervention.

## Figures and Tables

**Table 1:** Key genes and proteins involved in glycosylation that have been implicated in ASD through various lines of evidence.

Gene/Protein	Function in CNS	Type of Glycosylation	Nature of Evidence	Proposed Mechanism in ASD	Ref.
*NLGN4X*	Postsynaptic cell adhesion, synapse regulation	N-linked	High-penetrance mutations, functional studies	Impaired glycosylation leads to protein misfolding, reduced synaptic trafficking, and E/I imbalance	[47]
*B4GALT1*	Galactosyltransferase, glycan chain elongation	N- and O-linked	ASD susceptibility gene (GWAS), miRNA target hub	Altered glycosylation of cell surface receptors, affecting cell signaling and adhesion	[104]
*MAN2A2*	α-mannosidase, N-glycan maturation	N-linked	Compound heterozygous variants in ASD patient	Impaired N-glycan processing leads to accumulation of immature glycans, disrupting glycoprotein function	[5]
*EXT1*	Heparan sulfate biosynthesis	Glycosaminoglycan	CNVs in ASD, miRNA target	Disruption of neural ECM, affecting neuronal migration, axon guidance, and growth factor signaling	[104]
NCAM	Neuronal cell adhesion, synaptic plasticity	N-linked, Polysialylation	Polymorphisms, abnormal expression in ASD models	Altered cell–cell adhesion, impaired synaptic plasticity and connectivity	[29]
HNK1 Epitope	Glycan structure on multiple CAMs	N-linked (sulfated)	Functional studies in animal models	Absence impairs synaptic plasticity and learning; modifies CAM function, disrupting circuit formation	[59]
*GALNT9*	O-glycosyltransferase	O-linked	ASD susceptibility gene (GWAS)	Altered O-glycosylation of brain-specific proteins, function largely unknown	[105]

**Table 2:** Summary of distinct Glycosylation (enzymatic) and Glycation (non-enzymatic) biomarker candidates for ASD.

Biomarker Candidate	Biological Matrix	Analytical Method	Key Finding	Reported Diagnostic Accuracy/Significance	Ref.
I—Glycosylation markers					
Siaα2-3Gal/GalNAc-containing structures	Serum	Lectin Microarray	Significantly increased in ASD	Identified as a key differential glycopattern	[130]
Salivary Sialic Acid	Saliva	Not specified	Statistically lower in children with ASD	Correlated with expression of the *GNE* gene, a key enzyme in sialic acid biosynthesis	[105]
II—Glycation markers					
AGEs (CML, CMA, 3DG-H)	Plasma	LC-MS/MS	Increased in ASD	83% accuracy in children 5–12 years old	[131]
Methylglyoxal-derived adducts (MG-H1, CEL)	Plasma	LC-MS/MS	Increased in ASD	Positively correlated with ASD severity (ADOS-2 score)	[131]

## Data Availability

No new data were created or analyzed in this study.
